# Quantification of Left Ventricular Torsion and Diastolic Recoil Using Cardiovascular Magnetic Resonance Myocardial Feature Tracking

**DOI:** 10.1371/journal.pone.0109164

**Published:** 2014-10-06

**Authors:** Johannes T. Kowallick, Pablo Lamata, Shazia T. Hussain, Shelby Kutty, Michael Steinmetz, Jan M. Sohns, Martin Fasshauer, Wieland Staab, Christina Unterberg-Buchwald, Boris Bigalke, Joachim Lotz, Gerd Hasenfuß, Andreas Schuster

**Affiliations:** 1 Institute for Diagnostic and Interventional Radiology, Georg-August-University Göttingen, Göttingen, Germany; 2 Department of Computer Science, University of Oxford, Oxford, United Kingdom; 3 Papworth Hospital NHS Trust, Papworth Everard, Cambridgeshire, United Kingdom; 4 Children's Hospital and Medical Center, University of Nebraska College of Medicine, Omaha, Nebraska, United States of America; 5 Department of Pediatric Cardiology and Intensive Care Medicine, Georg-August-University Göttingen, Göttingen, Germany; 6 Department of Cardiology and Pneumology, Georg-August-University Göttingen, Göttingen, Germany; 7 Division of Imaging Sciences and Biomedical Engineering, The Rayne Institute, St. Thomas' Hospital, King's College London, London, United Kingdom; 8 DZHK (German Centre for Cardiovascular Research), partner site Göttingen, Göttingen, Germany; 9 Medizinische Klinik III, Kardiologie und Kreislauferkrankungen, Eberhard-Karls-Universität Tübingen, Tübingen, Germany; Northwestern University Feinberg School of Medicine, United States of America

## Abstract

**Objectives:**

Cardiovascular magnetic resonance feature tracking (CMR-FT) offers quantification of myocardial deformation from routine cine images. However, data using CMR-FT to quantify left ventricular (LV) torsion and diastolic recoil are not yet available. We therefore sought to evaluate the feasibility and reproducibility of CMR-FT to quantify LV torsion and peak recoil rate using an optimal anatomical approach.

**Methods:**

Short-axis cine stacks were acquired at rest and during dobutamine stimulation (10 and 20 µg·kg^−1^·min^−1^) in 10 healthy volunteers. Rotational displacement was analysed for all slices. A complete 3D-LV rotational model was developed using linear interpolation between adjacent slices. Torsion was defined as the difference between apical and basal rotation, divided by slice distance. Depending on the distance between the most apical (defined as 0% LV distance) and basal (defined as 100% LV distance) slices, four different models for the calculation of torsion were examined: Model-1 (25–75%), Model-2 (0–100%), Model-3 (25–100%) and Model-4 (0–75%). Analysis included subendocardial, subepicardial and global torsion and recoil rate (mean of subendocardial and subepicardial values).

**Results:**

Quantification of torsion and recoil rate was feasible in all subjects. There was no significant difference between the different models at rest. However, only Model-1 (25–75%) discriminated between rest and stress (Global Torsion: 2.7±1.5°cm^−1^, 3.6±2.0°cm^−1^, 5.1±2.2°cm^−1^, p<0.01; Global Recoil Rate: −30.1±11.1°cm^−1^s^−1^,−46.9±15.0°cm^−1^s^−1^,−68.9±32.3°cm^−1^s^−1^, p<0.01; for rest, 10 and 20 µg·kg^−1^·min^−1^ of dobutamine, respectively). Reproducibility was sufficient for all parameters as determined by Bland-Altman analysis, intraclass correlation coefficients and coefficient of variation.

**Conclusions:**

CMR-FT based derivation of myocardial torsion and recoil rate is feasible and reproducible at rest and with dobutamine stress. Using an optimal anatomical approach measuring rotation at 25% and 75% apical and basal LV locations allows effective quantification of torsion and recoil dynamics. Application of these new measures of deformation by CMR-FT should next be explored in disease states.

## Introduction

The prevalence of heart failure including systolic and diastolic functional impairments continues to increase representing a major health problem [1], [2]. Several diagnostic techniques have been proposed to study systolic and diastolic myocardial performances [3], [4], [5].

Left ventricular (LV) systolic torsion and diastolic recoil describe the myocardial twisting and untwisting motion resulting from apical counter-clockwise and basal clockwise rotation during systole (when viewed from the apex and normalized for LV length). Torsion and recoil rate have shown to be sensitive markers for systolic and diastolic dysfunction [6], [7], [8]. The phenomenon can be captured by the simple twist angle (difference in apical and basal rotation) [9], [10], the circumferential-longitudinal shear angle [11], [12] or torsion (twist per cardiac length) [13], [14]. Recent studies showed that torsion might represent an important compensatory mechanism to maintain an adequate systolic function in patients with chronic hypertension as examined in a large community-based population based on cardiovascular magnetic resonance (CMR) myocardial tagging [14], [15]. Recoil rate seems particularly appealing to study diastolic function. Its applicability has been demonstrated in isolated diastolic dysfunction [16], in heart failure with preserved ejection fraction [17] and heart disease characterized by both systolic and diastolic dysfunction such as hypertrophic cardiomyopathy (HCM) [18]. CMR myocardial tagging is considered the reference standard for the evaluation of torsion at the current time. However, this technique has not found widespread implementation into clinical routine since practical obstacles, e.g. the need for additional sequence acquisition and time-consuming post-processing limit its clinical applicability. Recent advances in LV deformation quantification based on CMR feature tracking (CMR-FT) allow straightforward quantification of myocardial physiology from routine steady-state free precession (SSFP) images [19], [20]. The technique has been used to analyse ventricular strain in health and disease [21], [22]. However, the feasibility of CMR-FT for quantitative assessment of LV torsion has never previously been demonstrated.

We therefore aimed to first develop a model that allows uniform selection of apical and basal slices at standardized LV levels and to evaluate its feasibility and reproducibility to quantify LV systolic torsion and diastolic recoil at rest and during dobutamine stress using CMR-FT.

## Materials and Methods

Ten healthy volunteers underwent CMR on a 1.5 Tesla scanner (Intera R 12.6.1.3, Philips Medical Systems, Best, The Netherlands). The study protocol was approved by the Institutional Review Board at the University of Nebraska Medical Center and complies with the Declaration of Helsinki. Written informed consent was obtained from all participants.

### CMR Imaging

All CMR measurements were performed in the supine position using a 5-channel cardiac surface coil. LV dimensions and function were assessed with an ECG- gated SSFP cine sequence during brief periods of breath-holding in 12 to 14 equidistant short-axis planes (slice thickness 8 mm; gap 1–2 mm) completely covering the LV. The field of view was 360×480 mm and matrix size 196×172. Dobutamine stress imaging was performed as previously described [23]. Complete short-axis stacks were acquired at rest and with 10 and 20 µg·kg^−1^·min^−1^ of dobutamine.

### Haemodynamic analysis

End-diastolic (EDV) and end-systolic volume (ESV), stroke volume (SV), ejection fraction and cardiac index (CI), were calculated using MassK-Mode in QMass Version 7.6 (Medis Medical Systems, Leiden, The Netherland). Ventricular volumes were adjusted to body surface area. All parameters were analysed at rest, 10 and 20 µg·kg^−1^·min^−1^ of dobutamine stress. Furthermore, heart rate and mean blood pressure were measured at rest and with both levels of dobutamine stimulation.

### Cardiac magnetic resonance myocardial feature tracking

Myocardial feature tracking was performed using dedicated software (TomTec Imaging Systems, 2D CPA MR, Cardiac Performance Analysis, Version 1.1.2.36, Unterschleissheim, Germany). All short-axis slices were included in the analysis between the most apical slice showing LV cavity at end-systole and the most basal slice including a complete circumference of myocardium at end-systole. LV endocardial and epicardial borders were manually traced in all slices using a point-and-click approach with the initial contour set at end-diastole. Endocardial and epicardial border surfaces were manually delineated and the automated tracking algorithm was applied. The software automatically tracks 48 subendocardial and subepicardial tissue voxels throughout the cardiac cycle. Tracking performance was visually reviewed to ensure accurate tracking of the ventricular myocardium. In case of insufficient border tracking manual adjustments were made to the initial contour and the algorithm was reapplied. Tracking was repeated for three times in each short-axis view and results were based on the average of the three repeated measures.

### Definition of torsion and recoil rate

**Figure 1:**
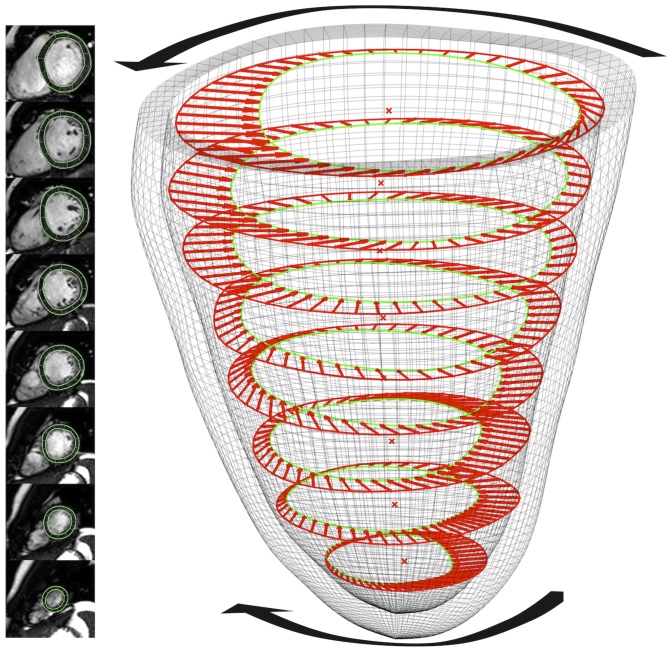
3 D model of LV rotational displacement. Rotation between time points (angular difference between red and green contours) is computed in each slice, and it is then linearly interpolated between slices. The 3D ventricular model is automatically fitted to the contours [48] and is used here only for illustration purposes, and not for the definition of the location of the most apical and basal points.

**Figure 2:**
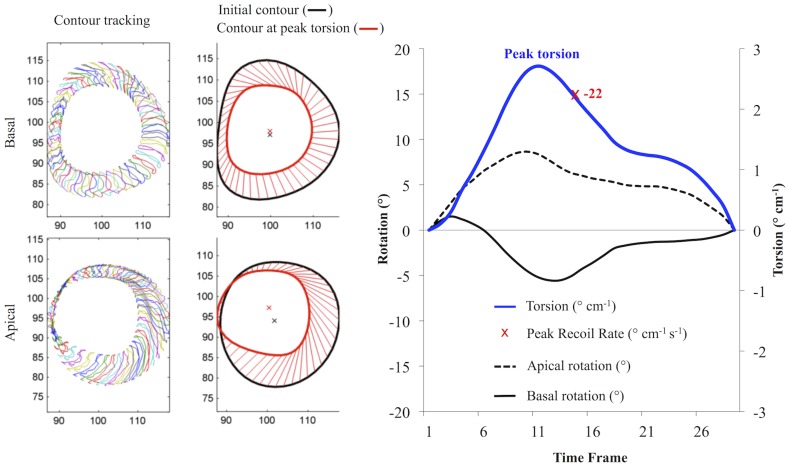
Evaluation of rotation. Rotational displacement (degrees) of 48 voxels was tracked throughout the cardiac cycle (**left**). Left ventricular torsion (° cm^−1^) was calculated as the difference in counter-clockwise (positive) apical rotation and clockwise (negative) rotation at the base, divided by the inter-slice distance (**right**).

**Figure 3:**
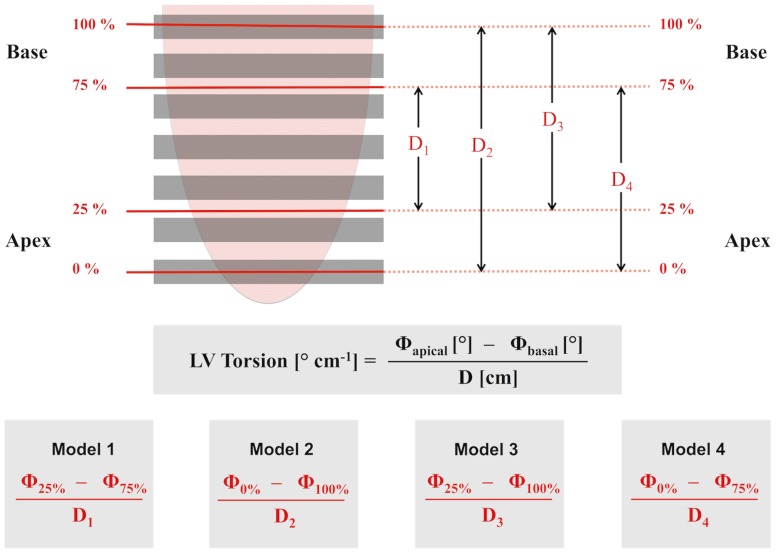
Definitions to calculate torsion. CMR feature tracking was performed in all slices of a short-axis stack. Four models (1–4) to calculate torsion were evaluated. Left ventricular (LV) torsion was calculated as the difference in counter-clockwise apical rotation (**φ**_apex_) and clockwise rotation at the base (**φ**_base_), divided by the inter-slice distance (D). The rotation of points at 0% (φ_0%_) and 100% (φ_100%_) distance correspond to the most apical and most basal levels. The rotation of points at 25% (φ_25%_) and 75% (φ_75%_) distance correspond to points that are typically located in between slices (linearly interpolated from adjacent slices). This approach allows generating constant distances (D_1_-D_4_) between corresponding apical and basal slices independently of varying cardiac anatomy.

Torsion and peak recoil rate were calculated using MATLAB software (The Math Works, MA, USA). Averaged LV rotation profiles were calculated from the angular displacement of all 48 tissue voxels in all slices. LV rotation was averaged across the three repeated measurements. The rotation of points that would typically be located in between adjacent slices was linearly interpolated resulting in a complete 3D LV model of angular displacement (**Figure 1**). When viewed from the apex, LV torsion was calculated as the difference in counter-clockwise apical rotation (**φ**_apex_) and clockwise rotation at the base (**φ**_base_) divided by the inter-slice distance (D) [13], [14]:
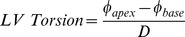



The distance (D) was calculated as the sum of the slice thickness (8 mm) and gap (1–2 mm) of imaging planes that were located in between the basal and apical slices where rotation was measured [14] (**Figure 2** **and** **3**). Depending on these measurement positions of rotation obtained at different LV locations and the distance between the most apical (defined as 0% of LV distance) and the most basal slice (defined as 100% of LV distance) the following four methods to calculate torsion were examined: Model 1 (difference in rotation between 25% and 75%), model 2 (difference in rotation between 0% and 100%), model 3 (difference in rotation between 25% and 100%) and model 4 (difference in rotation between 0% to 75%) (**Figure 3**). The analysis included subepicardial and subendocardial torsion as well as LV global torsion (averaged subendocardial and subepicardial torsion). Furthermore, subendocardial, subepicardial and global peak recoil rates were calculated from the maximum slope (−dθ/dt) of the diastolic limb of the torsion-time curve (**Figure 2**).

### Statistical Analysis

Statistical analysis was performed using Microsoft Excel and IBM SPSS Statistics version 22 for Macintosh. Data are expressed as mean (± standard deviation). Differences of torsion and peak recoil rate calculated between models 1–4 were assessed by the Friedman test. Pairwise comparisons between variables at rest and with increasing levels of dobutamine were evaluated using the Wilcoxon test. All statistical tests with p values <0.05 were considered statistically significant.

Two independent observers analysed all cases to assess inter-observer variability (JTK & AS). Intra-observer variability was derived from the repeated analysis by the first observer (JTK) after four weeks. The intra- and inter-observer variability were assessed by intraclass correlation coefficients (ICC) using a model of absolute agreement. Agreement was considered excellent when ICC>0.74, good when ICC = 0.60–0.74, fair when ICC = 0.40–0.59, and poor when ICC<0.4 [24]. Furthermore, Bland Altman analysis [25] and coefficients of variation (CoV) were calculated. The CoV was defined as the standard deviation of the differences divided by the mean [26].

## Results

The image quality was sufficient to perform CMR-FT in all subjects and for all slices at rest and during dobutamine stress. CMR-FT post-processing took 10 to 12 minutes for a LV short-axis stack on average including adjustments if necessary. Participant demographics are summarised in **table 1**.

**Table 1:** Subject characteristics.

Demographics	Healthy volunteers
Study population	N = 10
Gender (f/m)	5/5
Age (y)	40.6 (23–51)
LV-EDV (ml/m^2^)	51.0 (7.5)
LV-ESV (ml/m^2^)	21.7 (5.1)
LV-CI (l/min/m^2^)	2.0 (0.4)
LV-EF (%)	57.9 (5.6)

Continuous variable are expressed as mean (standard deviation), age is expressed as median (range).

f: female, m: male, EDV: end-diastolic volume, ESV: end-systolic volume, CI: cardiac index, EF: ejection fraction.

### Haemodynamic response to dobutamine

There were no side effects to dobutamine exposure. There was a significant increase of cardiac output, mean blood pressure and ejection fraction between rest and both levels of dobutamine as well as between 10 and 20 µg·kg^−1^·min^−1^ of dobutamine. Stroke volume increased significantly between rest and both levels of dobutamine, whilst no further increase was observed between 10 and 20 µg·kg^−1^·min^−1^ of dobutamine (**table 2**).

**Table 2:** Volumetric and hemodynamic response to 10 and 20 µg kg^−1^min^−1^ of dobutamine.

Parameter	Level of dobutamine (µg kg^−1^ min^−1^)			P value		
	Rest	10	20	Rest vs. 10	Rest vs. 20	10 vs. 20
LV-CI (l/min/m^2^)	2.0 (0.4)	3.3 (0.8)	4.2 (0.6)	**<0.01**	**<0.01**	**<0.01**
LV-EDV (ml/m^2^)	51.0 (7.5)	52.7 (9.1)	43.8 (15.4)	0.33	**<0.05**	**<0.01**
LV-ESV (ml/m^2^)	21.7 (5.1)	14.4 (5.9)	11.4 (4.6)	**<0.01**	**<0.01**	**<0.01**
LV-SV (ml/m^2^)	29.4 (4.1)	38.3 (7.4)	37.3 (6.5)	**<0.01**	**<0.01**	0.24
LV-EF (%)	57.9 (5.6)	72.9 (9.5)	77.0 (5.7)	**<0.01**	**<0.01**	**<0.05**
Mean BP (mmHg)	91.5 (10.2)	98.6 (10.4)	102.9 (10.7)	**<0.01**	**<0.05**	**<0.05**
Heart Rate	69.3 (10.3)	85.2 (16.6)	112.8 (11.9)	**<0.01**	**<0.01**	**<0.01**

Bold p values indicate a significance level <0.05.

BP: blood pressure, other abbreviations as in table 1.

### Torsion and recoil rate at rest

Inter-slice distances used for torsion calculation were 2.9±0.4 cm (model 1), 5.8±0.7 cm (model 2) and 4.4±0.5 cm (model 3 and 4). There was no significant difference of endocardial, epicardial or global torsion and endocardial and global recoil rate between the models 1–4 at rest. Results are displayed in **table 3**. There was a significant difference (p = 0.02) of epicardial recoil rate between model 2 (0–100%) and model 4 (0–75%).

**Table 3:** Torsion and recoil rate.

	Model 1 (25–75%)	Model 2 (0–100%)	Model 3 (25–100%)	Model 4 (0–75%)	P value
**Torsion Endo (°/cm)**	3.0 (1.9)	3.6 (1.6)	3.6 (1.7)	3.4 (2.0)	0.72
**Torsion Epi (°/cm)**	2.3 (1.3)	2.7 (0.9)	2.6 (1.4)	2.6 (0.8)	0.37
**Global Torsion (°/cm)**	2.7 (1.5)	3.2 (1.2)	3.1 (1.5)	3.0 (1.3)	0.56
**Recoil Rate Endo (°/cm/s)**	−34.2 (10.3)	−46.2 (18.3)	−42.2 (9.7)	−45.5 (24.7)	0.56
**Recoil Rate Epi (°/cm/s)**	−26.1 (14.6)	−26.4 (9.3)	−24.8 (12.5)	−31.4 (10.6)*	**0.048**
**Global Recoil Rate (°/cm/s)**	−30.1 (11.1)	−36.3 (12.7)	−33.5 (9.1)	−38.5 (16.4)	0.29

Baseline values for torsion and recoil rate in comparison between the four models.

**Variables are given as mean (SD)**

*p = 0.022 vs. Model 2

Bold p values indicate a significance level <0.05.

Endo: subendocardial, Epi: subepicardial, Global: average of subendocardial and subepicardial, rest: measurement at rest, 10: level of 10 µg kg^−1^ min^−1^ of dobutamine, 20: level of 20 µg kg^−1^ min^−1^ of dobutamine.

### Torsion and recoil rate during dobutamine stress

**Table 4** summarises torsion and recoil rate parameters at rest and during dobutamine stress as calculated by the different models. The mean inter-slice distance during dobutamine exposure was slightly lower compared with rest due to increased longitudinal shortening with stress. The inter-slices distances were 2.8±0.3 cm (model 1), 5.6±0.6 cm (model 2) and 4.2±0.4 cm (model 3 and 4) for measurements during 10 and 20 µg·kg^−1^·min^−1^ of dobutamine.

**Table 4:** Comparison of torsion and recoil rate between rest and stimulation with dobutamine.

		Level of dobutamine (µg kg^−1^min^−1^)			P value		
		rest	10	20	Rest vs. 10	Rest vs. 20	10 vs. 20
**Model 1 (25**–**75%)**	**Torsion Endo (°/cm)**	3.0 (1.9)	4.6 (2.5)	6.0 (2.4)	**0.02**	**<0.01**	**0.02**
	**Torsion Epi (°/cm)**	2.3 (1.3)	2.7 (1.5)	4.1 (2.2)	0.33	**0.01**	**0.03**
	**Global Torsion (°/cm)**	2.7 (1.5)	3.6 (2.0)	5.1 (2.2)	0.06	**<0.01**	**0.01**
	**Recoil Rate Endo (°/cm/s)**	−34.2 (10.3)	−53.6 (14.8)	−79.1 (35.1)	**0.01**	**<0.01**	**0.04**
	**Recoil Rate Epi (°/cm/s)**	−26.1 (14.6)	−40.3 (19.2)	−58.6 (32.3)	**0.02**	**<0.01**	**0.04**
	**Global Recoil Rate (°/cm/s)**	−30.1 (11.1)	−46.9 (15.0)	−68.9 (32.3)	**<0.01**	**<0.01**	**0.03**
**Model 2 (0**–**100%)**	**Torsion Endo (°/cm)**	3.6 (1.6)	5.2 (2.5)	5.8 (2.5)	0.14	0.09	0.51
	**Torsion Epi (°/cm)**	2.7 (0.9)	3.8 (2.0)	3.9 (1.8)	0.29	0.06	0.45
	**Global Torsion (°/cm)**	3.2 (1.2)	4.5 (2.2)	4.8 (1.9)	0.17	0.07	0.51
	**Recoil Rate Endo (°/cm/s)**	−46.2 (18.3)	−72.7 (37.9)	−82.6 (32.9)	0.17	0.047	0.33
	**Recoil Rate Epi (°/cm/s)**	−26.4 (9.3)	−49.0 (34.0)	−55.5 (28.0)	0.047	**<0.01**	0.39
	**Global Recoil Rate (°/cm/s)**	−36.3 (12.7)	−60.8 (34.7)	−69.1 (28.3)	0.11	**0.02**	0.39
**Model 3 (25**–**100%)**	**Torsion Endo (°/cm)**	3.6 (1.7)	4.8 (2.6)	6.1 (3.0)	0.07	**<0.01**	0.09
	**Torsion Epi (°/cm)**	2.6 (1.4)	2.9 (1.4)	3.9 (2.1)	0.51	**0.02**	**0.03**
	**Global Torsion (°/cm)**	3.1 (1.5)	3.8 (2.0)	5.0 (2.4)	0.07	**<0.01**	**0.02**
	**Recoil Rate Endo (°/cm/s)**	−42.2 (9.7)	−62.9 (29.7)	−69.5 (25.0)	**0.02**	**0.02**	0.45
	**Recoil Rate Epi (°/cm/s)**	−24.8 (12.5)	−37.8 (18.5)	−48.3 (25.3)	**0.02**	**0.01**	0.07
	**Global Recoil Rate (°/cm/s)**	−33.5 (9.1)	−50.4 (23.6)	−58.9 (24.6)	**0.01**	**0.01**	0.33
**Model 4 (0**–**75%)**	**Torsion Endo (°/cm)**	3.4 (2.0)	5.4 (2.5)	5.5 (3.0)	0.17	0.11	0.96
	**Torsion Epi (°/cm)**	2.6 (0.8)	4.0 (2.3)	4.1 (1.7)	0.24	**0.02**	0.20
	**Global Torsion (°/cm)**	3.0 (1.3)	4.7 (2.3)	4.8 (1.8)	0.14	**0.047**	0.88
	**Recoil Rate Endo (°/cm/s)**	−45.5 (24.7)	−69.8 (39.4)	−99.5 (45.6)	0.24	**0.01**	0.11
	**Recoil Rate Epi (°/cm/s)**	−31.4 (10.6)	−55.0 (34.7)	−70.4 (38.4)	0.06	**<0.01**	0.14
	**Global Recoil Rate (°/cm/s)**	−38.5 (16.4)	−62.4 (36.4)	−84.9 (38.9)	0.09	**0.01**	0.11

Bold p values indicate a significance level <0.05. Abbreviations as in table 3 *.*

**Figure 4:**
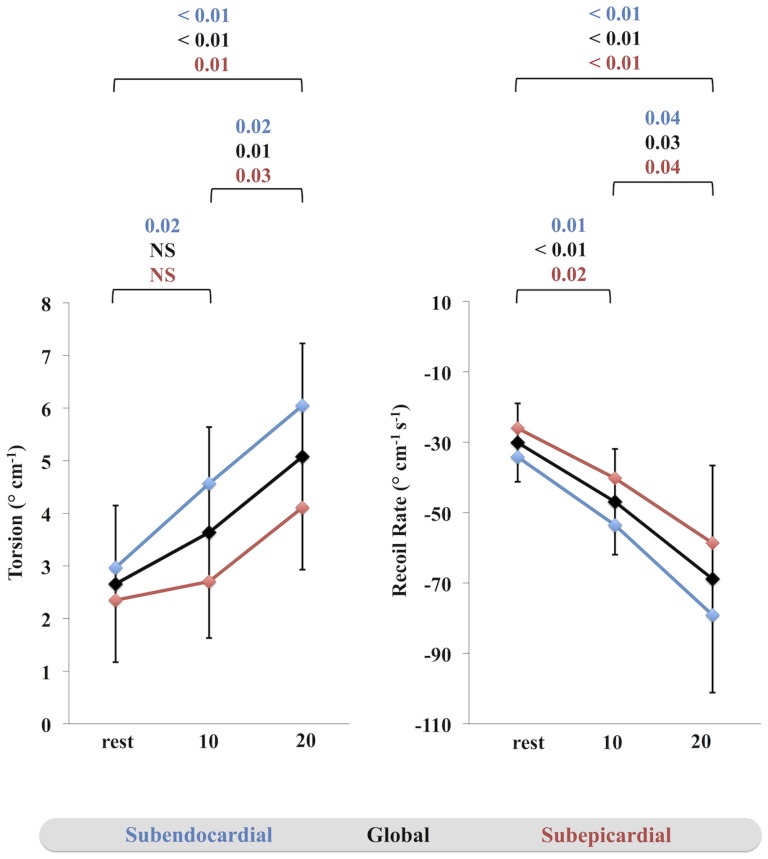
Torsion and recoil rate during dobutamine stress. Torsion and recoil rate (blue: subendocardial; red: subepicardial, black: global) as derived using model 1 (25–75%) in comparison between rest and increasing levels of dobutamine. A p-value <0.05 was considered statistically significant.

Model 1 (25–75%) was the only approach that revealed significantly increased torsion and recoil rate (subendocardial, subepicardial and global) between rest and both levels of dobutamine as well as between 10 and 20 µg·kg^−1^·min^−1^ of dobutamine except there was no significant difference of subepicardial torsion (p = 0.33) and global torsion (p = 0.06) between rest and 10 µg·kg^−1^·min^−1^ of dobutamine (**Figure 4**). The increase of torsion during dobutamine stress was more pronounced on the subendocardial as compared to the subepicarial level. All other models showed only partly significant results or trends towards increased torsion and recoil rates as outlined in **table 4**.

### Reproducibility

Bland Altman analysis, ICC and CoV are displayed in **table 5**. There was no difference in reproducibility between rest and dobutamine stimulation. ICC for model 1 (25–75%) and model 3 (25–100%) consistently showed values above 0.85. The model 2 (0–100%) and 4 (0–75%) showed slightly higher inter-observer variability for subendocardial torsion with ICC of 0.78 and 0.79, respectively.

**Table 5:** Reproducibility for all four models to calculate torsion and diastolic recoil rate.

		Model 1 (25–75%)			Model 2 (0–100%)			Model 3 (25–100%)			Model 4 (0–75%)		
		Mean Difference (SD)	ICC (95%CI)	CoV (%)	Mean Difference (SD)	ICC (95%CI)	CoV (%)	Mean Difference (SD)	ICC (95%CI)	CoV (%)	Mean Difference (SD)	ICC (95%CI)	CoV (%)
**Intra-observer**	**Torsion Endo (°/cm)**	0.02 (1.36)	0.92 (0.83–0.96)	30.14	−0.06 (0.79)	0.97 (0.94–0.99)	16.09	−0.16 (0.83)	0.97 (0.95–0.99)	16.91	0.00 (1.04)	0.96 (0.91–0.98)	21.80
	**Torsion Epi (°/cm)**	−0.02 (0.91)	0.94 (0.87–0.97)	29.81	0.05 (0.42)	0.99 (0.97–0.99)	12.23	0.03 (0.51)	0.98 (0.95–0.99)	16.50	−0.02 (0.61)	0.97 (0.94–0.99)	16.99
	**Global Torsion (°/cm)**	0.00 (1.05)	0.93 (0.86–0.97)	27.65	−0.01 (0.50)	0.98 (0.96–0.99)	12.07	−0.06 (0.49)	0.99 (0.97–0.99)	12.14	−0.01 (0.66)	0.97 (0.94–0.99)	15.81
	**Recoil Rate Endo (°/cm/s)**	6.48 (15.24)	0.92 (0.82–0.96)	25.88	0.98 (23.49)	0.91 (0.81–0.96)	34.73	−2.82 (10.88)	0.95 (0.90–0.98)	19.17	−0.56 (31.59)	0.88 (0.75–0.95)	44.29
	**Recoil Rate Epi (°/cm/s)**	−0.44 (9.74)	0.96 (0.92–0.98)	23.50	−0.81 (11.72)	0.95 (0.90–0.98)	27.11	−1.64 (7.51)	0.96 (0.92–0.98)	20.79	−3.80 (16.43)	0.93 (0.85–0.97)	32.63
	**Global Recoil Rate (°/cm/s)**	3.02 (9.68)	0.96 (0.92–0.98)	19.30	0.09 (15.77)	0.94 (0.87–0.97)	28.44	−2.23 (7.89)	0.96 (0.92–0.98)	16.97	−2.18 (21.34)	0.91 (0.82–0.96)	35.08
**Inter-observer**	**Torsion Endo (°/cm)**	0.07 (1.58)	0.89 (0.77–0.95)	35.30	0.94 (1.33)	0.85 (0.54–0.94)	30.35	0.52 (1.18)	0.94 (0.85–0.97)	25.84	0.62 (1.89)	0.80 (0.59–0.91)	42.31
	**Torsion Epi (°/cm)**	−0.15 (0.74)	0.96 (0.92–0.98)	23.55	0.35 (0.78)	0.94 (0.85–0.97)	23.73	0.21 (0.50)	0.98 (0.94–0.99)	16.54	0.14 (1.03)	0.93 (0.85–0.97)	29.61
	**Global Torsion (°/cm)**	−0.04 (1.07)	0.93 (0.86–0.97)	28.04	0.64 (0.97)	0.89 (0.68–0.96)	25.33	0.37 (0.70)	0.96 (0.91–0.99)	18.44	0.38 (1.35)	0.86 (0.71–0.93)	33.78
	**Recoil Rate Endo (°/cm/s)**	8.09 (17.07)	0.90 (0.76–0.95)	28.60	−9.31 (17.17)	0.91 (0.77–0.96)	27.47	−0.70 (8.61)	0.97 (0.94–0.99)	14.89	−7.01 (25.30)	0.89 (0.76–0.95)	37.15
	**Recoil Rate Epi (°/cm/s)**	−0.05 (9.42)	0.96 (0.93–0.98)	22.63	−5.35 (12.64)	0.93 (0.85–0.97)	30.85	−2.35 (7.02)	0.97 (0.93–0.98)	19.61	−6.42 (16.56)	0.92 (0.82–0.96)	33.77
	**Global Recoil Rate (°/cm/s)**	4.02 (10.76)	0.95 (0.90–0.98)	21.24	−7.33 (10.86)	0.95 (0.82–0.98)	21.00	−1.52 (6.02)	0.98 (0.96–0.99)	12.86	−6.71 (17.38)	0.92 (0.83–0.96)	29.67

ICC: Intraclass-correlation coefficient; CoV: coefficient of variation; SD: standard deviation; CI: confidence interval. Other abbreviations as in table 3.

## Discussion

Myocardial twisting and untwisting represent fundamental mechanisms of LV systolic and diastolic function. It has been suggested that even up to 40% of stroke volume is produced from systolic twisting forces [27]. The subsequent fast untwisting during early diastole is a major indicator of the restoring forces that contribute to rapid diastolic filling [28], [29]. In a clinical context, a simple, quick and robust quantification of LV twisting and untwisting motion is therefore desirable. The present study applied CMR-FT to assess both systolic torsion and diastolic recoil directly from routine SSFP cine images to capture these phenomena and demonstrates several important findings. CMR-FT based quantification of myocardial twisting motion as assessed with torsion and diastolic recoil is feasible and reproducible. Furthermore, we were able to show that CMR-FT allows discrimination of both systolic torsion and diastolic recoil at rest and dobutamine stress when using standardized analyses based on rotation measurements at 25% and 75% LV distance.

### Feasibility of CMR-FT derived torsion

The presented approach used a complete LV rotational model to define exact apical and basal slice positions by assessing rotational profiles of all short-axis slices of a short-axis stack with subsequent linear interpolation of inter-slice rotation between adjacent slices. This approach appears suitable since several studies revealed strict linear progression of rotation from base to apex [10], [12], [14]. Previous reports have shown that the amount of LV rotation is highly dependent on the site of measurements at apical and basal levels. This needs to be taken into account when calculating the simple twist angle [10]. We therefore applied torsion (twist normalized to inter-slice distance) to quantify the myocardial twisting motion since this technique is supposed to be less influenced by the variation in rotation as compared to the simple twist definition (difference in apical and basal rotation) [30]. The results of our study indicate, that in fact CMR-FT quantification of torsion at rest is not that dependant on measurement positions since we have not detected any significant differences between the four examined models that used rotation from different standardized LV levels serving for the calculation of torsion. However, differences in recoil rate were more pronounced. Hence, quantification of recoil rate might be more dependent on the exact slice position as compared to quantification of torsion. This is particularly important when quantification of diastolic function is required which underpins the need for a method allowing a uniform slice selection at standardized LV levels. It is important to note that our methodology allows accurate, specific assessments of LV torsion and diastolic recoil at different levels throughout the myocardial wall which could be particularly valuable in the assessment of myocardial diseases that predominantly affect the endocardial layer such as subendocardial myocardial infarction, cardiac amyloid or endomyocardial fibrosis.

### Reproducibility of CMR-FT derived torsion

CMR-FT represents a relatively novel approach to quantify myocardial deformation. Previous CMR-FT studies primarily focused on ventricular strain measurements. The reported amount of reproducibility and repeatability varies between studies with most studies reporting reasonable reproducibility of global strain levels [19], [31], [32], [33]. Therefore, intra- and inter-observer variability for torsion and recoil rate needs to be taken into special consideration. In order to maximise reproducibility all measurements were repeated three times and corresponding averaged rotational profiles were included in the calculation of torsion. As a result, all torsion and recoil rate parameters were highly reproducible on an intra-observer and inter-observer level. We found slightly lower reproducibility of model 2 (0–100%) and model 4 (0–75%) as compared to model 1 (25–75%) or model 3 (25–100%). Both, model 2 and model 4, involve rotational measurements taken from the most apical slice included in the calculation of torsion. As described in previous reports, reproducibility for CMR-FT derived strain parameters was lower at apical myocardial levels than at mid-ventricular or basal levels [34]. Our study shows that this holds true for apical and basal rotation assessment with CMR-FT and emphasises to rather include apical rotation from a slice position at 25% LV distance instead of using the most apical slice of a short-axis stack.

### Torsion as a function of dobutamine exposure

Previous studies investigated the effect of inotropic stimulation with dobutamine on LV torsion in a canine model and found increased torsion paralleled by accelerated recoil rate [35], [36]. The findings were confirmed in a human model using echocardiographic speckle tracking [37]. The authors found that subepicardial torsion remained unchanged during low dose dobutamine infusion and increased with higher doses of dobutamine. In contrast, subendocardial torsion already increased during low dose dobutamine infusion [37]. In the present study, we showed that model 1 (25–75%) using the shortest distance between apical and basal slices, consistently revealed significant increases of subendocardial and global torsion as well as accelerated recoil rate (subendocardial, subepicardial and global) with increasing levels of dobutamine. Conversely, subepicardial torsion only increased with the application of an intermediate dose of dobutamine, which is in accordance with the published findings by Akagawa et al. [37]. The other models using either the most apical or basal slice did not reliably differentiate torsion and recoil rate between rest and pharmacological stress and only showed non-significant trends towards increased torsion and recoil rates. This is most likely explained by through-plane motion causing loss of tracked features from the imaging plane. This might be particularly pronounced in the very apical and basal LV slices since longitudinal shortening is increased during dobutamine stress and may explain the need to measure torsion from positions a bit farer away from the apex and the base.

### CMR-FT in the context of existing methods to calculate torsion

Previously described CMR based modalities for the study of myocardial twisting and untwisting motion include myocardial tagging [10], [38], tissue phase mapping [39] and displacement encoding with stimulated echoes (DENSE) [40]. Although accelerated techniques have been reported for myocardial tagging [41], [42], all mentioned approaches commonly require additional sequence acquisitions and are usually associated with time-consuming post-processing both limiting their clinical applicability. In contrast, CMR-FT allows direct quantification of myocardial motion from standard SSFP images, which are widely available since they are generally part of every clinical CMR volumetric examination. From a clinical perspective, evaluation of myocardial torsion using CMR-FT has the potential to be integrated into a given clinical CMR protocol. Speckle-tracking echocardiography is another technique that has proved feasible for the quantification of myocardial rotation [8], [10], [43]. Speckle tracking echocardiography is widely available and benefits from high temporal resolution but suffers from limited image quality especially occasionally poor acoustic windows [23]. In general echocardiography does not allow a reliable and uniform selection of apical and basal slices in individual subjects, which has proved to be crucial for a robust quantification of myocardial twisting motion as expressed by our data and in previous reports [10]. Furthermore it is challenging to quantify torsion (twist per length) with speckle-tracking echocardiography, since the assessment of the inter-slice distance between apical and basal slices is generally not possible. Speckle-tracking echocardiography is therefore limited to the simple twist definition, which is even more dependent on the exact slice locations and difficult to reproduce in longitudinal studies [30]. Notwithstanding these considerations, several echocardiography speckle tracking based studies have shown the feasibility of twisting and untwisting motion quantification in various heart diseases. These involve conditions with a predominant diastolic involvement such as heart failure with preserved ejection fraction [17], diseases with predominant systolic involvement such as ischemic cardiomyopathy [44] and also diseases that equally affect systole and diastole such as hypertrophic cardiomyopathy [45] or cardiac amyloidosis [46]. Whether or not such assessments could also be derived from CMR-FT torsion and diastolic recoil measurements need to be investigated with future research studies.

Regarding longitudinal assessments, previous studies applied myocardial tagging to investigate the inter-study reproducibility for quantitative analysis of torsion and recoil rate and found good reproducibility for torsion whilst peak recoil rate was only poorly reproducible [38]. In fact, the study of recoil rate may require a more strict definition of measurement positions at apical and basal levels. This may be challenging using myocardial tagging, since this is usually performed at 3 LV levels only (apical, mid-ventricular and basal) and therefore subject to manual planning of slice positions. In contrast, we have now introduced a CMR-FT based technique that allows a uniform and exact definition of apical and basal slice positions independent of different heart sizes. Future work will need to address whether this approach positively impacts on inter-study reproducibility of torsion and of recoil rate in particular.

### Limitations

The sample size of this feasibility study was relatively small. Notwithstanding this fact the results demonstrate feasibility and good reproducibility for torsion and diastolic recoil quantification with CMR-FT from routine cine images. Ideally we would have compared our results to myocardial tagging representing the reference standard for such acquisitions. However, given the necessity to obtain a whole SSFP short-axis stack at rest and with different levels of dobutamine we felt that the acquisition of an equal number of tagged images would have resulted in scanning times difficult to tolerate during dobutamine stimulation.

A general limitation of CMR based quantification of myocardial twisting motion is the current lack of standardized methods. Future studies will need to compare CMR-FT derived torsion and recoil rate with parameters from myocardial tagging using equal computation (twist per length) [38] or alternative computational methods e.g. the circumferential-longitudinal shear angle [11], [12] which has not been addressed in the present study. However, there is evidence to suggest that twisting mechanics as assessed by torsion (twist per length) and shear angle deliver somewhat similar results [15]. The individual merits of both methods remain to be addressed in future investigations. A further limitation of both, conventional myocardial tagging and CMR-FT is the two-dimensional acquisition technique. We developed a pseudo three-dimensional mesh of myocardial torsion based on linear interpolation. However, this method does not compensate for through plane motion. Previous studies compared 2D and 3D derived torsion measured by myocardial tagging [47] and found that through plane motion did not significantly impact on 2D torsion since both methods showed reasonable agreement. The authors concluded to rather use the faster and easier 2D method for calculation of LV torsion. If this also holds true for 2D derived torsion from CMR-FT remains to be addressed in future investigations.

The clinical applicability of CMR-FT derived torsion and diastolic recoil assessment will depend on the speed of the analysis. At the present time the result files originating from CMR-FT are analysed using an in-house module for MATLAB. Future software developments will need to incorporate both post-processing steps in a single application to further minimize post-processing time.

Finally the current work aimed to determine the feasibility and reproducibility to quantify torsion and recoil rate from CMR-FT in a collective of healthy volunteers at rest and during dobutamine stress. Future research needs to prospectively validate this novel technique in pathologies such as coronary artery disease, heart failure or valvular disease.

## Conclusions

CMR-FT allows derivation of myocardial torsion directly from standard SSFP cine images. Torsion and recoil rate calculated from apical and basal slices with 25–75% distance consistently discriminated between rest and increasing levels of dobutamine. Reproducibility was good to excellent for all torsion and recoil rate parameters, but was especially high when using an apical level at 25% LV distance instead of the most apical slice. When using CMR-FT to quantify myocardial torsion, it is therefore suggested to select apical and basal slices at standardized 25% and 75% levels. The analysis of myocardial torsion and recoil motion based on CMR-FT may have potential clinical and research applications when exact quantification of systolic and diastolic function is required. Future validation studies in larger cohorts should follow to establish the clinical utility and the prognostic implications of this technique.
